# Influence of aprepitant and localization of the patch on fentanyl exposure in patients with cancer using transdermal fentanyl

**DOI:** 10.18632/oncotarget.24812

**Published:** 2018-04-06

**Authors:** Evelien J.M. Kuip, Wendy H. Oldenmenger, Martine F. Visser-Thijs, Peter de Bruijn, Esther Oomen-de Hoop, Ron H.J. Mathijssen, Carin C.D. Van der Rijt, Stijn W. Koolen

**Affiliations:** ^1^ Department of Medical Oncology, Erasmus MC Cancer Institute, Rotterdam, The Netherlands; ^2^ Department of Medical Oncology, Radboud University Medical Center, Nijmegen, The Netherlands; ^3^ Department of Anesthesiology, Pain and Palliative Care, Radboud University Medical Center, Nijmegen, The Netherlands

**Keywords:** fentanyl, aprepitant, pharmacokinetics, cancer

## Abstract

**Background and Objectives:**

The cutaneous fentanyl patch is widely used to treat continuous pain in patients with cancer. Its use is hampered by a high inter- and intrapatient pharmacokinetic variability. Factors that influence this pharmacokinetic variability are largely unclear. The aim of these studies was to test if common patient variables, i) the use of the moderate CYP3A4 inhibitor aprepitant and ii) the localization of the fentanyl patch (upper arm versus thorax) influence systemic exposure to fentanyl in patients with cancer using a transdermal fentanyl patch.

**Results:**

The AUC_0–6 h_ of fentanyl was 7.1% (95% CI: −28% to +19%) lower if patients concurrently used aprepitant, compared to the period when patients used fentanyl only. The AUC_0–4 h_ of fentanyl was 7.4% (95% CI: −22% to +49%) higher when the cutaneous fentanyl patch was applied to the upper arm compared to application at the thorax.

**Conclusions:**

Neither the concurrent use of aprepitant, nor the localization of the fentanyl patch showed a statistically significant influence on fentanyl pharmacokinetics.

**Methods:**

We performed two prospective cross-over pharmacokinetic intervention studies. Both studies had two eight-day study periods. At day 8 of each study period blood samples were collected for pharmacokinetic analysis. In each study 14 evaluable patients were included.

## INTRODUCTION

Since decades the fentanyl cutaneous patch is used to treat chronic cancer pain [1]. The patch is widely used mainly because of its patient-friendly administration route [2, 3]. This patch is applied to the skin and has to be changed every 72 hours/3 days. Fentanyl is absorbed through the intact skin and forms a subcutaneous depot. Absorption is mediated by diffusion and is influenced by the thickness of the lipophilic keratinous *stratus corneum* [4, 5]. When fentanyl passes through the skin, fentanyl is absorbed into the microcirculation followed by the systemic circulation [1, 4].

A steady state is usually reached after application of a second transdermal fentanyl patch [6], although plasma concentration vary over the 72 hour period wherein a single patch is used [7]. Unfortunately, there is a wide intra- and interpatient pharmacokinetic variation in patients using fentanyl patches [7–11]. In clinical practice patients may already describe less painkilling effects of the cutaneous patch after 48 hours, and they may use extra opioids in the last 24 hours. Or they need to change their cutaneous patch every 48 hours leading to extra costs which are not always reimbursed by the insurers company. Despite the fact that numerous factors have been investigated, this variation is still largely unexplained [8]. The area under the curve (AUC) of fentanyl increased up to 3-fold in volunteers who used strong CYP3A4 inhibitors (like troleandomycine or ritanovir) together with fentanyl [12–16]. The combination of the moderate CYP3A4 inhibitor fluconazole and fentanyl showed a significant decrease in clearance of fentanyl [15].

Patients with cancer commonly require polypharmacy to treat side effects of (chemo-) therapy, complications of the underlying cancer or other diseases. Pharmacokinetic drug-drug interactions in cancer patients are therefore highly relevant [17, 18]. This is further emphasized by two case reports describing severe and even lethal fentanyl intoxications after a drug-drug interaction between fentanyl and fluconazole or itraconazole, respectively [19, 20]. Further study on the concurrent use of CYP3A4 inhibitors and fentanyl is therefore warranted. Aprepitant is deemed a moderate CYP3A4 inhibitor. It is the first neurokinin-1 (NK-1) receptor antagonist and it is used in combination with a 5-hydroxytryptamine-3 (5HT_3_) antagonist and dexamethasone for the prevention of nausea and vomiting in case of highly or moderately emetogenic chemotherapy [21, 22]. Both aprepitant and fentanyl are thus widely and simultaneously used in cancer patients and because of aprepitant's inhibitory capacity on CYP3A4, it could hypothetically increase the exposure of fentanyl, leading to more side effects like nausea or sleepiness. Nonetheless, higher systemic fentanyl concentrations could also lead to a better control of pain. Nevertheless, clinicians should always be aware of potential drug-drug interactions with fentanyl and more frequently monitor pain and side effects in these patients unexplained [8].

Another factor that may influence fentanyl exposure is the localization of the patch on the skin. Now, a fentanyl patch is advised to be applied on dry, intact, skin of the trunk, upper arm, or leg. Most patients prefer the upper arm. When changing the patch, it always has to be applied at another place because of the subcutaneous depot. However, also the localization where the fentanyl patch is applied may influence fentanyl absorption due to differences in skin thickness and/or the amount of subcutaneous fat. Two previous studies measured the residue in used patches of patients with cancer. Comparison of 100 patients showed a 7.5% lower delivery efficiency of fentanyl for patches applied to the leg in comparison to the arm [23]. The other study showed no differences in fentanyl absorption between patches applied to arm, shoulder, chest and back [11]. However, in both studies plasma fentanyl concentrations were not measured and both studies used inter patient comparisons, making the conclusions less robust given the high interpatient variation mentioned above.

We hypothesized that because fentanyl is highly lipophilic, higher plasma concentrations will be reached when the patch is used on areas with thicker skin, as they usually contain more fat. Mean skin thickness of the upper arm and the upper back are almost equal (43.9 μm versus 43.4 μm),while the mean skin thickness of the thorax is less (37.6 μm) [24]. Therefore, we expected differences in fentanyl concentrations between the upper arm/ upper back and the ventral thorax region for sticking the fentanyl patch. For convenience of the patient we choose to compare the upper arm with thorax region for the transdermal delivery of fentanyl in the current study.

In this report we describe the results of these two studies in which the effect of the concomitant use of aprepitant and the localization of the patch on the exposure to fentanyl were investigated.

## RESULTS

### Aprepitant study

A total of 20 patients was included, while 6 patients were withdrawn from the study before start of PK sampling because of a deteriorated condition. As a result, 14 patients (6 females and 8 males) with a median age of 61 years (IQR 55–71) completed the study and were evaluable. Unfortunately, two patients had missing PK measurements at the 6 hour time point. The demographic data of the evaluable patients are presented in Table 1.

**Table 1:** Patient characteristics in aprepitant study

	*N* = 14		
Sex, n		
Male	8	
Female	6	
Age in years (median and IQR)	60.5 (55–71)	
Height in cm (median and IQR)	172.5 (167–180)	
Weight in kg (median and IQR)	71 (67–92)	
BMI (median and IQR)	26 (19.5–29.5)	
Fentanyl patch dose (μg/h) mean (range)	25 (12–43.5)	
Laboratory results (n = 12) (median (IQR) (normal range)	Period 1	Period 2
Creatinine (55–90 μL/min)	67 (62–92)	67 (61–86)
MDRD ( > 60 mL/min/1,73 m2)	87 (66–90)	85 (70–90)
AST ( < 31 U/L) (n = 11)	30 (20–47)	26 (21–45)
ALT ( < 34 U/L)	23 (14–41)	24 (12–40)
Bilirubin ( <17 umol/L)	7 (4–12)	8 (4–12)
ALP (< 98 U/L) (n = 11)	133 (87–282)	133 (90–247)

No significant differences were found in the chemistry results between period 1 and 2, and therefore did not affect the outcomes of the study.

**Figure 1:**
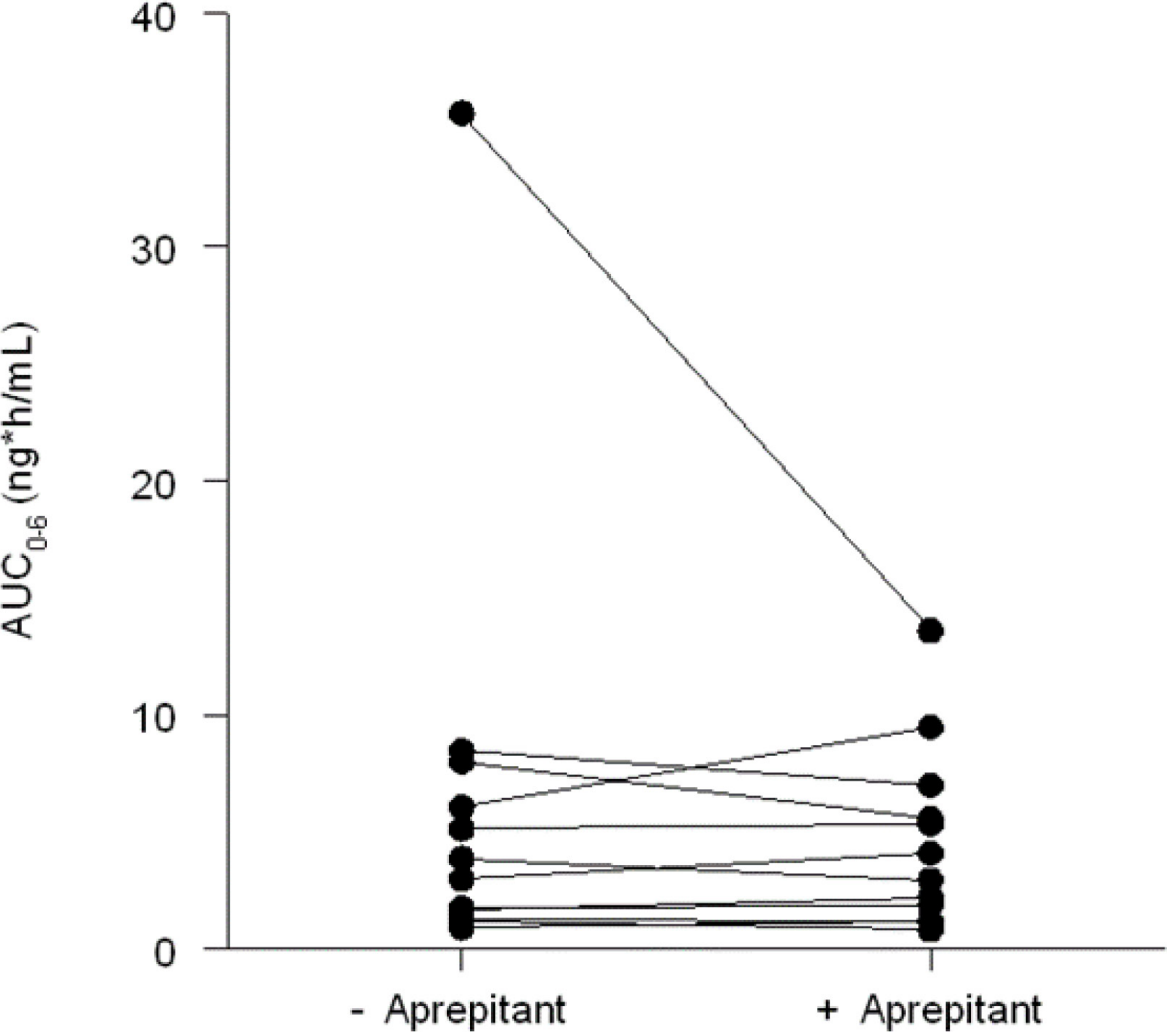
Plasma fentanyl concentrations (AUC_0–6 h_) aprepitant (right) versus no aprepitant (left)

The AUC_0–6 h_ was 7.1% (95% CI: −28% ; +19%) lower when fentanyl was used in combination with aprepitant as compared to using fentanyl without aprepitant. The inter- and intra-patient coefficients of variation in fentanyl were 59% and 28%, respectively. Log-transformed fentanyl concentrations are shown in Figure 1. AUC_0–4 h_ analysis was also performed and showed the same non-significant results (relative difference in AUC_0–4 h_ was 4.5% (95% CI: −24%; +20%, fentanyl with aprepitant in comparison to fentanyl without aprepitant).

### Patch localization study

**Figure 2:**
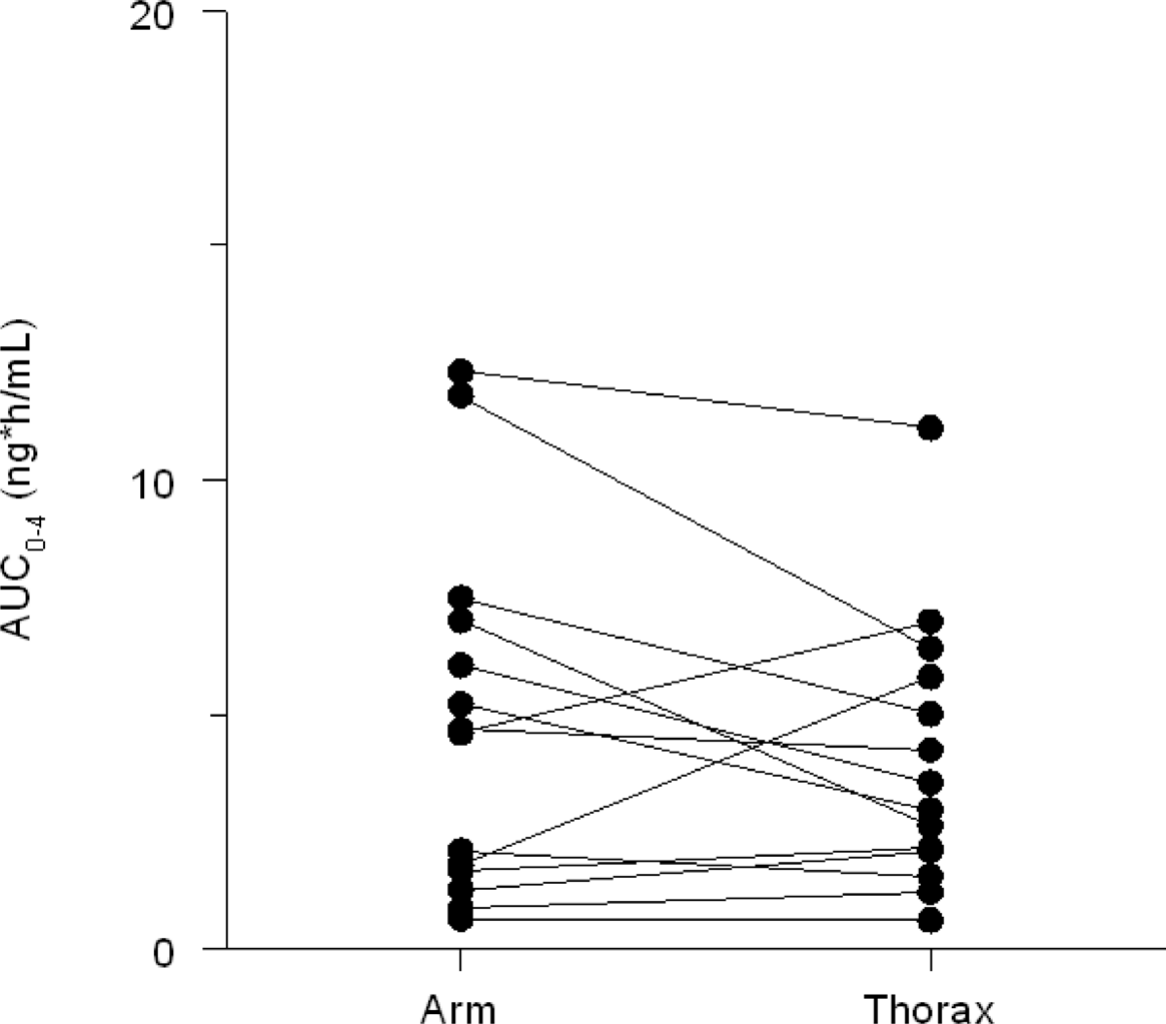
Plasma fentanyl concentrations (AUC_0–4 h_) upper arm vs thorax

Twenty-three patients were included. Fourteen patients (11 females and 3 males) with a median age of 62 years (IQR 57–65) completed the study and were evaluable. The demographic information about these patients is presented in Table 2. The other nine patients were not evaluable due to clinical deterioration and missed blood sampling for pharmacokinetic analyses. The AUC was 7.4% (95% CI: −22% ± 49%) higher when the patch was applied to the upper arm as compared to the thorax. The inter- and intra-patient coefficient of variation in fentanyl (normalized AUC) were 48% and 41%, respectively Figure 2.

**Table 2:** Patient characteristics in patch localization study

	*N* = 14
Sex, n	
Male	3
Female	11
Age, years (median and IQR)	62 (57–65)
Height, cm (median and IQR)	167 (162–172)
Weight, kg (median and IQR)	66 (63–78)
BMI (median and IQR)	23.6 (22.6–28.0)
Fentanyl patch dose (μg/h) mean (range)	52.5 (12–175)

## DISCUSSION

The concomitant use of aprepitant for 3 days showed no statistically significant influence on the AUC of fentanyl in patients with cancer using transdermal fentanyl. Neither did the localization of the fentanyl patch.

Several studies investigated drug-drug interactions with aprepitant before, because of its ability to inhibit CYP3A4 [25]. However only one study investigated drug-drug interactions with aprepitant and opioids [26]. Concomitant use of aprepitant and oxycodone in patients with cancer led to a 25% higher AUC of oxycodone [26]. Therefore, it is surprising that a drug like aprepitant, that is highly metabolized by CYP3A4 did not increase the exposure of fentanyl in a clinically and statistically significant extent. To limit the number of sampling moments in our patients, we have limited our study to just 1 course aprepitant of 3 days. The effect of multiple courses would have been interesting.

Studies, as mentioned in the introduction, with strong CYP3A4 inhibitors did result in increased fentanyl exposure, although the effect sizes in those reports were smaller than theoratically expected [8, 12, 14, 16]. A previous study with fluconazole, a moderate CYP3A4 inhibitor like aprepitant, showed a significantly lower clearance of fentanyl (11.6 ± 3 mL/min/kg vs 14.0 ± 2.5 mL/min/kg) when used together, but no significant difference on AUC. In our study only AUC was measured, so possible effects on other pharmacokinetic parameters are unknown.

The most accepted hypothesis of fentanyl metabolism is that fentanyl is mainly metabolized in the liver by CYP3A4 mediated N-dealkylation resulting in the inactive metabolite norfentanyl [4, 27, 28]. However, a recent study showed that other unknown metabolic routes might also play a role in fentanyl metabolism and that the N-dealkylation step might be less predominant than previously thought [16], thereby possibly explaining the limited influence of aprepitant on fentanyl exposure. In that study, the metabolic clearance of fentanyl to norfentanyl was strongly inhibited by ketoconazole, but only a small increase of fentanyl exposure in general was seen [16]. For future research it would be interesting to study the different metabolites in plasma and urine to see whether aprepitant has an impact on the formation of those metabolites.

We studied only the combination of aprepitant with transdermally applied fentanyl. Several rapid onset forms of fentanyl are now available, and we cannot exclude that there will be an effect of aprepitant on these formulations.. Previous studies with CYP3A4 inhibitors and fentanyl used mostly intravenously administrated fentanyl [14–16, 29, 30]. Of the rapid onset opioids only transmucosal fentanyl citrate has been studied [28]. The combination of the strong CYP3A4 inhibitor troleandomycine showed in both intravenously and transmucosally administrated fentanyl higher AUC's compared to fentanyl alone [28, 29]. Since effect sizes of CYP3A inhibitors may be different among various administration routes, the results of the current analysis cannot be extrapolated to rapid onset opioids. Therefore, extra attention is needed when aprepitant is prescribed to patients who also use fentanyl rapid onset opioids .

In this study the localization of the fentanyl patch did not statistically significantly influence the exposure to fentanyl. An interpatient comparison in another study investigating fentanyl delivery, by analyzing patches, between patients applying patches to the leg versus the thorax found a small non-significant 7.5% difference in favor of the arm [23]. Our intra-patient comparison showed a similar (non-significant) difference between the arm and the upper thorax. Unfortunately, actual skin thickness or other characteristics describing skin condition were not measured in our study. Despite that, our study describes the situation in daily clinical care and is therefore of relevance for both patients and physicians. This study demonstrated that skin thickness is of minor importance for transdermally delivered fentanyl.

The inter individual variation in plasma fentanyl levels were much larger than we had expected. Therefore our studies were underpowered to find a clinically and statistically significant difference of 30% in the AUC’s.

## CONCLUSIONS

In these two cross-over studies we could not identify any effect of aprepitant or the localization of the patch on fentanyl pharmacokinetics.

## METHODS

The two studies were performed as single-center pharmacokinetic studies at the Erasmus MC Cancer Institute. Inclusion criteria were similar for the two studies: patients with cancer, age ≥ 18 years, using a stable dose of a transdermal fentanyl (Durogesic^®^) for at least 8 days irrespective of the dose used and given written informed consent. Exclusion criteria were: use of fentanyl rescue medication (other opioids were allowed), the use of strong CYP inhibitors or inducers [31] and evidence of serious psychiatric illness, confusion or intellectual disability.

### Aprepitant study

**Figure 3:**
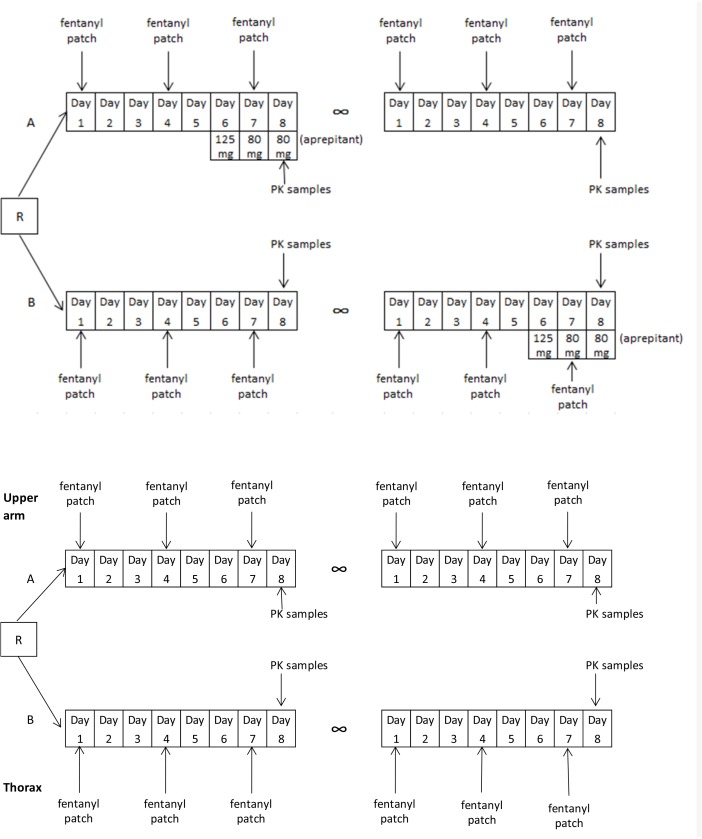
Study design of both the aprepitant study (upper scheme) and patch localization study (bottom scheme)

This study used a randomized cross-over design with two study periods, each lasting eight days. In both periods patients used a stable dose of fentanyl, whereas patients were randomized for the use of aprepitant between arm A and arm B (Figure 3). Patients in arm A used aprepitant in the first study period, whereas patients in arm B used aprepitant in the second study period. Patients applied the patch alternately to the right and left upper arm, with a new patch on day one of each study period. The patch was changed every 3 days (72 hours), according to label instructions. Aprepitant was used in the order: 125 mg-80 mg-80 mg on day 6, 7 and 8 of the study period, respectively, concurrently with the fentanyl patch. Pharmacokinetic sampling was performed at day 8, approximately 24 hours after changing a patch. Venous blood samples were taken at baseline (just before taking aprepitant) and at 2, 3, 4, 5 and 6 hours after administration of aprepitant or at similar times for the periods in which aprepitant was not used. Blood samples were collected in potassium EDTA coated tubes.

The following routine chemistry data were collected: aspartate aminotransferase (AST), alanine aminotransferase (ALT), bilirubin, albumin and alkaline phosphatase (ALP), creatinine, estimated glomerular filtration rate (eGRF), calculated by Modification of Diet in Renal Disease (MDRD); formula: eGFR (mL/min/1.73 m^2^) = 32788 × serum Creatinine (μmol/L) −1.154 × age (years) −0.203 × (0.742 when female) × (1.210 when of African descent).

### Patch localization study

This study used a randomised cross-over design with two eight-day study periods as well. According to randomisation patients applied the patch to the upper arm (group A) or thorax (group B) first. The patch was changed every 3 days to the opposite arm or thorax, according to regular use. Pharmacokinetic sampling was performed at day 8, approximately 24 hours after changing a patch. Three venous blood samples were collected, with 2 hours between each sample. After collection of the blood samples patients switched to the other patch localization, either thorax or upper arm dependent on randomization. The same sampling procedure as during the first study period was followed.

### Measurements of fentanyl plasma concentrations

We quantified fentanyl in EDTA plasma. A validated UPLC-MS/MS method. This method consisted of a Waters Acquity UPLC sample manager, coupled to a triple quadruple mass spectrometer operating in the multiple reaction monitoring mode (MRM) with positive ion electro spray ionization (Waters, Etten-Leur, The Netherlands). The multiple reaction monitoring transitions was set at 337→188.

Chromatographic separations for fentanyl were achieved on an Acquity UPLC^®^ BEH C18 1.7 μm 2.1 × 100 mm column eluted at a flow-rate of 0.350 mL/min on a gradient of methanol. A cycle time for this method was about 6 minutes. Calibration curves were linear over a wide range (0.100 to 10.0 ng/mL) with at lower limit of quantitation (LLQ) of 0.100 ng/mL for fentanyl. The within and between-run precisions, including the LLQ, were ≤ 5.52 % and ≤ 6.12 %, respectively, while the average accuracy ranged from 86.2 % to 97.5%. The extraction of 200 μL of plasma involved a deproteinization step with acetone, followed by a simple liquid extraction with ethyl acetate. The organic phase was evaporated and subsequently dissolved in 100 μL methanolic solutions, from which aliquots of 10 μL were injected into the UPLC-MS/MS system.

### Statistics

For both studies (1. the combination of aprepitant and 2. the localization of the fentanyl patch) a difference in systemic exposure to fentanyl of 30% was determined to be clinically relevant. It was assumed that the intra-patient relative standard deviation in fentanyl pharmacokinetics was 20%. Given a power of 80%, 14 patients were required in each study to detect a difference. For the primary endpoint, the following analysis approach was taken. A natural log transformation was applied to the AUC_0–4 h_ and AUC_0–6 h_ values in order to normalize the distributions [4, 7, 32]. Estimates for the mean differences in (log) AUCs were obtained using a linear mixed effect model with treatment, sequence and period as fixed effects and subject within sequence as a random effect [33]. Variance components were estimated based on restricted maximum likelihood (REML) methods and the Kenward-Roger method of computing the denominator degrees of freedom was used. The mean differences and 95% CIs for the differences were exponentiated to provide point estimates of the ratio of geometric means and 95% CIs for these ratios, which can be interpreted as relative differences in percentages. Regular chemistry results, which were measured only in the aprepitant study, were compared between periods by means of the Wilcoxon signed rank test.

### Compliance with ethical standards

The two studies were approved by the medical ethics review board (aprepitant study; MEC 13.387 and patch localization study; MEC 12.193) and conducted in accordance with the Declaration of Helsinki. The trials were registered in the Dutch Trial Register (Trial registration ID: aprepitant study: NTR4288; localization study NTR3654). Written informed consent was obtained from all patients.
